# A predictive model and a field study on heterogeneous slug distribution in arable fields arising from density dependent movement

**DOI:** 10.1038/s41598-022-05881-w

**Published:** 2022-02-10

**Authors:** Sergei Petrovskii, John Ellis, Emily Forbes, Natalia Petrovskaya, Keith F. A. Walters

**Affiliations:** 1 School of Computing and Mathematical Sciences, University of Leicester, Leicester, UK; 2 Peoples Friendship University of Russia (RUDN University), 6 Miklukho-Maklaya St, Moscow, 117198 Russia; 3 The Pirbright Institute, Woking, GU24 0NF UK; 4 School of Mathematics, University of Birmingham, Birmingham, UK; 5 Centre for Integrated Pest Management, Harper Adams University, Newport, UK

**Keywords:** Animal migration, Ecological modelling, Agroecology

## Abstract

Factors and processes determining heterogeneous (‘patchy’) population distributions in natural environments have long been a major focus in ecology. Existing theoretical approaches proved to be successful in explaining vegetation patterns. In the case of animal populations, existing theories are at most conceptual: they may suggest a qualitative explanation but largely fail to explain patchiness quantitatively. We aim to bridge this knowledge gap. We present a new mechanism of self-organized formation of a patchy spatial population distribution. A factor that was under-appreciated by pattern formation theories is animal sociability, which may result in density dependent movement behaviour. Our approach was inspired by a recent project on movement and distribution of slugs in arable fields. The project discovered a strongly heterogeneous slug distribution and a specific density dependent individual movement. In this paper, we bring these two findings together. We develop a model of density dependent animal movement to account for the switch in the movement behaviour when the local population density exceeds a certain threshold. The model is fully parameterized using the field data. We then show that the model produces spatial patterns with properties closely resembling those observed in the field, in particular to exhibit similar values of the aggregation index.

## Introduction

A fundamental issue in ecology is the understanding of how system properties observed on a large spatial scale (field, landscape) emerge from processes operating on a much smaller scale^1^. An important example is given by spatial population distribution. Such distributions are often remarkably heterogeneous even in a relatively uniform environment; a phenomenon known as patchiness^2,3^. One well-established theory^4–7^ relates it to the interplay between the local population growth (resulting from individual births and deaths) and the population dispersal (e.g. resulting from individual movement) where the latter usually occurs on a spatial scale much larger than the former. The corresponding modelling framework extensively used in literature is based on the Turing theory of pattern formation, which was originally developed as a model of morphogenesis^8^ and later applied in the ecological context^9,10^.

While the Turing theory and its generalisations were shown to work well for vegetation patterns^7,11,12^, animal populations rarely exhibit spatial regularity typical for the Turing patterns. Contrary to plants, there are factors that severely restrict its application to spatial distribution of animal populations. One such factor is the ability of animals to move and the complexity of behavioural responses associated with this^13^—in particular, sociability, which results in collective behaviour. The collective behaviour is inherent in many species leading to the formation of animal groups (such as fish schools, bird flocks, insect swarms, etc.)^14^ and is a major factor responsible for the small-scale heterogeneity in animal spatial distribution. While the collective behaviour has many forms, reasons and implications (e.g., efficient defense against predators), perhaps it most obviously manifests itself in the context of movement^15^. Many animal groups are highly mobile and tend to move as a whole, with individual animal velocities strongly correlated^16^.

There are, however, species that exhibit a prominent small-scale spatial heterogeneity not explicitly related to group movement. In particular, distribution of soil invertebrates is often strongly heterogeneous^17^, one well studied example being given by the grey field slug (*Deroceras reticulatum*). The grey field slug is a typical invertebrate species in the UK, Europe and North America. It is an important part of the corresponding food webs, in particular being a food source for a wide range of predators and parasitic organisms from several major taxonomic groups. The species is also known to be a common pest in agricultural environments in many global regions, where it can cause considerable economic damage. Evidence for the grey field slug population distribution in natural environments is meagre, but in farm fields its distribution is known to exhibit a distinctly patchy structure on the subfield scale of 10–50 m^18^. Moreover, patches of high slug density were shown to be relatively stable within a season (with the spatial location of most patches showing little or no change) but not necessarily between seasons^18,19^, although precise reasons for the latter are not well understood. Earlier studies^20,21^ detected correlations between the slug density and a number of edaphic characteristics such as soil organic matter content, soil texture and others. However, heterogeneity of soil properties alone cannot explain the variation in slug density in a farm field environment, particularly where their spatial uniformity may be increased by some agronomic activities. More likely, high slug density patches emerge due an interplay between the environmental properties and behavioural mechanisms.

In this study, we show that the formation and stability of the patchy slug distribution may be a consequence of the specifics of individual slug movement. Such movement is shown to be density dependent: when con-specifics are close by, an individual slug moves differently compared to when it is alone^22^. It seems intuitive to expect that this type of movement density dependence may contribute to the patch stability^23^, although any quantitative evidence is lacking. It is much less clear whether it can lead to patch formation. Here we explore these issues employing a simulation model where the individual slug movement is parameterized using radio-tracking data collected in the field^22,23^. We show that the density dependence in individual slug movement indeed results in the formation of distinct patchy distributions with the statistical properties (quantified by the Taylor index) in good agreement with field observations.

## Summary of empirical findings

**Figure 1:**
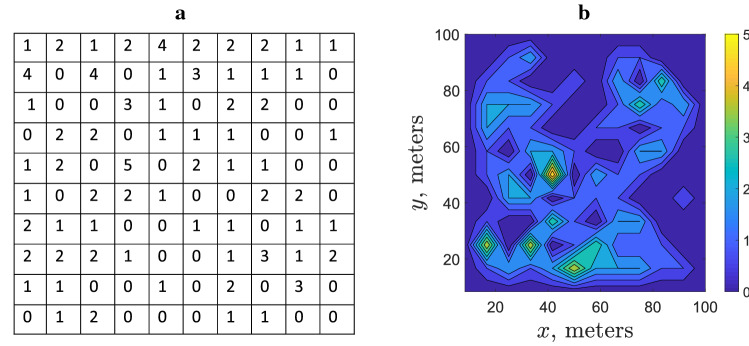
Example of a heterogeneous slug distribution. (**a**) Trap counts on the grid of traps recorded on 02 September 2016 in a crop field in Shropshire UK (see^18^ for full details), and (**b**) a proxy of the corresponding density distribution reconstructed from the trap counts using linear interpolation.

Spatial distribution of slugs in arable fields was studied intensively in a recent 3 year project^24^. In order to collect the data on slug abundance, square grids of 10 × 10 refuge traps (with 10 m distance between neighbouring traps) were set up in various commercial crop fields across England. Traps were checked and the counts recorded on a regular basis (every 2–3 weeks)^18^. Analysis of the field data collected in 167 sampling visits to the sites where traps were installed has shown that, apart from rather rare exceptions (linked to cases of very low average population density) slug distribution is distinctly heterogeneous, usually consisting of patches of high density separated by areas with very low slug density^18^. A typical example is shown in Fig. 1. The degree of aggregation was assessed using Taylor’s Power Law^25,26^. The corresponding index of aggregation (i.e. the exponent in the power law relating the variance and the mean of the sample) was shown to be consistently larger than 1, which indicated that the observed patchiness in the slug distribution cannot be reduced to purely statistical reasons. The patches of high slug abundance appear to be relatively stable, often (albeit not always) preserving their location for several weeks. In several cases, trap counts were collected on a finer grid with 2 m spacing between the traps. In some of those cases, trap counts exhibited a clear and significant variation in space^24^, indicating that pattern formation occurs on multiple scales.

In other related work, movement of individual slugs was studied using radio-tracking. Slugs were collected from the study field and radio-tagged in the laboratory (see^18,24^ for details) before being returned to the same field in two different release types: ‘sparse’ and ‘dense’. In the sparse release treatment, 17 slugs were released individually at locations separated by 2 m distance. In the dense release treatment, 11 slugs were released as a group, i.e. were placed within a small domain of approximately 0.1 m$^2$. Radio-tagging enabled individual slugs to be uniquely identified and their movement accurately tracked for extended time periods, and consecutive locations of all released slugs were then checked and recorded over a 10 hour observational period with a defined time interval^23^. The movement data were analysed using the discrete-time random walk framework^27–30^ that parameterizes the movement with frequency distributions for its three essential components: the spatial step size, the turning angle, and the proportion of movement/resting time. It has been found in^23^ that all three movement components are significantly different following the sparse and dense releases, with the general tendency for slug movement to be faster and with longer distances (per unit time) covered in each step in the sparse case, i.e. in the absence of con-specifics in their vicinity.

## Methods

Movement of slugs is simulated using a discrete-time individual-based model (IBM), which is defined and parameterized using the field data from a recent study; see^23^ for all details of the paramater estimation procedure. In order to account for the observed density-dependence, the classical IBM approach (e.g.^31^) is modified as follows. On top of the three movement characteristics, i.e. the movement/rest time ratio, the turning angle and the step length (each of them quantified by a probability distribution), we add two special parameters. First, we introduce a perception radius $R \ge 0$, i.e. the maximum distance in any direction that an individual can detect the presence of other animals^32,33^. Based on the results of the field study, the value of *R* is estimated for the grey field slug as $0.2\le R\le 2$ m. Second, we introduce the density threshold *d*. If the slug density $D_l$ within the individual's perception radius is below the threshold, the slug’s move to its next position is calculated using the characteristics for the ‘sparse movement’. If the slug density $D_l$ is above the density threshold, then calculations use ‘dense movement’ characteristics. From the available field data, the density threshold is roughly estimated as $0.25\le d\le 100$ (slugs/m$^{-2}$), i.e., the movement is known (see “Summary of empirical findings” section) to be density dependent when the slug density is 100 (in the above units) and density independent when the density is 0.25.

Consider a population of *N* slugs, where the location of the *n*th slug ($n=1,2,\ldots ,N$) at a given time $t=0,1,2,\ldots$ is given by $(x_n(t),y_n(t))$. In order to calculate the position of each individual slug at time $t+1$, we first decide whether it moves as in dense movement or in sparse movement. For this purpose, $D_l$ is calculated (by counting all individuals within *R* and dividing the number by $\pi R^2$) and compared to *d*.

We then decide whether the slug will move at all or remain at the same position. Let $P_m$ be the average movement frequency i.e., the ratio of the number of time steps in which slug movement is recorded to the total number of time steps registered in observations. For each slug at each time step, we assume that movement occurs when $p < P_m$, where the probability of movement *p* is drawn from a uniform distribution in the interval [0, 1]. Importantly, $P_m$ is different for the dense and sparse movement, that is

1 $$\begin{aligned} P_m = P_m^s~~~\text{ if }~~D_l<d \quad \text{ and }\quad P_m = P_m^d~~~\text{ if }~~D_l \ge d. \end{aligned}$$

The average movement frequencies $P_m$ that slug movement data showed in the sparse and dense releases were evaluated^23^ as $P_m^s \approx 0.5$ for sparse movement and $P_m^d \approx 0.25$ for dense movement.

In the case where the slug is going to move, we then define the movement direction $\theta$. This is quantified by the turning angle, i.e. the angle made between the directions of the previous and the next steps. Based on the analysis of field data^23^, the probability distribution for the turning angle is as follows. For slugs in the sparse movement, we use a Von Mises distribution:

2 $$\begin{aligned} \rho (\theta )=\frac{\exp (\kappa \cos (\theta -\mu ))}{2\pi I_0(\kappa )}. \end{aligned}$$

Analysis of slug movement data provided the parameter values in (2) as $\mu =0$ and $\kappa =0.8$^23^. For slugs in the dense movement, description of the data by a Von Mises distribution yields the value $\kappa \approx 0$; correspondingly, for the turning angle distribution we use the uniform distribution:

3 $$\begin{aligned} \rho (\theta )=\frac{1}{2\pi }, \end{aligned}$$

(which is the limiting case of Von Mises distribution in the case $\kappa =0$).

Finally, the value of the step length $\Delta r$ is calculated based on its probability distribution. For the slug movement, the latter was shown^23^ to be best described by a half-normal distribution both for the sparse and dense movement but with a different variance:

4 $$\begin{aligned} \rho _G(\Delta r|0,\sigma ^2)=\frac{2}{\sqrt{2\pi \sigma ^2}}\exp \left( -\frac{(\Delta r)^2}{2\sigma ^2}\right) , \end{aligned}$$

where

5 $$\begin{aligned} \sigma ^2 = \sigma ^2_s~~~\text{ if }~~D_l<d \quad \text{ and }\quad \sigma ^2 = \sigma ^2_d~~~\text{ if }~~D_l \ge d. \end{aligned}$$

The distribution (4) has been fitted to the data to obtain $\sigma _s=0.105$ m for sparse movement and $\sigma _d=0.113$ m for dense movement over a given time interval of, on average, 30 minutes.

We emphasize that the expressions in (1–5) do not contain any free parameter, as all parameter values are determined from the existing field data.

Once all the above calculations are done, the position of the slug at time $(t+1)$ is simulated as

6 $$\begin{aligned} \left( x_n(t+1),y_n(t+1)\right) =(x_n(t)+\Delta x, y_n(t)+\Delta y), \end{aligned}$$

where

7 $$\begin{aligned} \Delta {x} =(\Delta {r})\cos (\theta ), \quad \Delta {y} =(\Delta {r})\sin (\theta ), \end{aligned}$$

provided the slug moves rather than remaining at rest, as is quantified by the corresponding probabilities, see Eq. (1).

For convenience, we consider the non-dimensional time $t=t^*/\Delta t^*$ where $t^*$ is the real time in minutes and $\Delta t^*=30$ min is a typical experimental time interval, so that $\Delta t=1$ in the model equates to 30 minutes. However, we keep dimensional units for the length, so that the spatial scale of the emerging spatial patterns can be readily seen. We perform simulations in two different spatial domains or ‘fields’: a small field of $10\times 10$ m (consistent with the field data on individual slug movement, see^23^) and a large field of $100\times 100$ m, the latter being directly comparable to the spatial domain in which data on slug abundance were collected^18^.

The movement process (6–7) starts from an initial condition—the initial position for all slugs, i.e., $(x_n(0),y_n(0))$. In most cases we use a statistically uniform spatial distribution where the coordinates $x_{n}(0)$ and $y_{n}(0)$ ($n=1,2,\ldots ,N$) of the *n*-th slug at time $t=0$ are random numbers drawn from a uniform distribution.

## Results

### Patch formation

**Figure 2:**
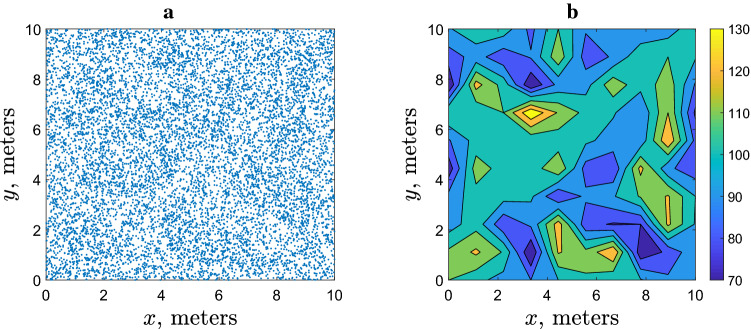
Example of simulation results and their visualization obtained at $t=100$ for $R=1$ (meters) and the total number of slugs $N=10^4$ in a $10\times 10$ m field. The chosen threshold density is equal to the average slug density, i.e. $d=100$ (slugs/m$^{-2}$). Other movement parameters are given in the text, see the lines after Eqs. (1), (3) and (5). (**a**) Positions of all individual slugs, (**b**) the corresponding density distribution reconstructed from the bin counts (see details in the text) using linear interpolation.

The individual-based simulations produce, as an immediate result, the position of all slugs at any given time *t*; an example is given in Fig. 2a in the case of ‘small’ field $10\times 10$ m. Analysing information describing the system state when it is presented in this format can be difficult. It is a particular problem when comparing it with field data where the system state is quantified by the slug trap counts (regarded as a proxy for the local slug density) at selected spatial locations, e.g. in a rectangular grid (cf. Fig. 1a). We therefore split the spatial domain into 100 bins by dividing its linear size both in *x* and *y* into 10 equal intervals and calculate the number of slugs in each bin, i.e. the population density at the location of each bin. In order to make the results more accessible for the visual perception, we then show the binned numbers as a continuous plot of the population density. As an example, Fig. 2b shows the distribution of the population density corresponding to the simulation data shown in Fig. 2a.

**Figure 3:**
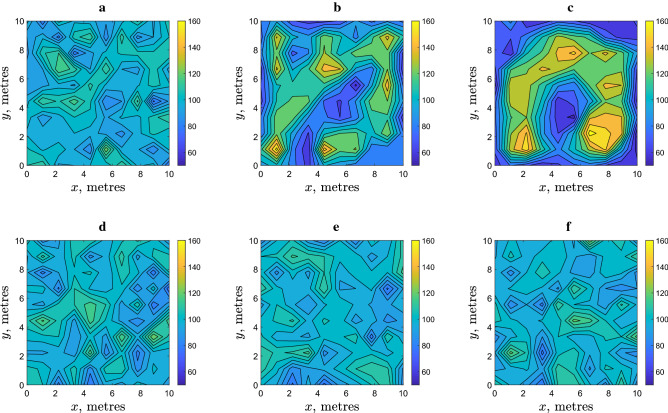
The spatial distribution of $N=10^4$ slugs shown at different moments of time: (**a**,**d**) $t=0$, (**b**,**e**) $t=10^3$, (**c**,**f**) $t=10^4$. Distributions in the upper row (**a**–**c**) are obtained using the density dependent movement model (as is described in “Methods” section). Parameter values are the same as in Fig. 2. For comparison, the lower row (**d**–**f**) shows the distributions obtained in the case of density independent movement. While patches of high density (shown by yellow colour) emerge in the course of time in the case of density dependent movement, they do not emerge in the case of purely random, density independent movement.

We readily observe that the simulated spatial distribution of slugs is apparently heterogeneous. Two questions immediately arise here as to (i) whether this heterogeneity is different from the heterogeneity of a purely statistical origin and (ii) how the density distribution evolves in time. To answer these questions, Fig. 3b,c show the simulation results after a series of increasing time periods using the same initial condition (Fig. 3a) used for Fig. 2. It is readily seen that the distribution evolves with time and the degree of heterogeneity (e.g. as described by the difference between the smallest and the largest values of the population density, inferred from the numbers on the colour bar) tends to increase over the course of time resulting in the formation of high density patches (shown by the yellow colour). Also, the size of individual patches changes, with a tendency to increase until it reaches a certain value (see the next section for details). For comparison, Fig. 3e,f show the spatial distribution obtained at the same moments of time as in Fig. 3b,c respectively, but in case of purely random density-independent movement; no high density patches emerge in that case.

Simulations show that the emerging high density patches are dynamic rather than stationary (even in the large-time limit, for more details, see Appendix A.2 in online Supplement Information). No stationary distribution emerges in the course of time. However, inspection of the results shown in Figs. 2b and 3b,c (as well as results obtained in other simulation runs, not shown here for the sake of brevity) reveals that the patch dynamics is rather slow, so that some of the patches roughly preserve their size and location on the timescale of $t=10^4$, i.e. about 3 weeks in dimensional units, which is in a good agreement with the field data, see Section 2.

Note that, since our model is inherently stochastic, the emerging spatial distribution will differ in the precise shape and position of the patches between model runs. However, the formation of a distinct patchy structure is a generic property of the system. In this sense, the patterns shown in Fig. 3b,c are typical for the system’s dynamics. Moreover, the formation of the patchy structure appears to be robust and does not depend on the initial conditions. For example, in the case where the initial condition is chosen as a dense release (i.e., all animals are initially inside a single patch), over the course of time the initial patch eventually splits into a number of smaller patches resulting, for the same parameter values as in Fig. 3, in a spatial distribution qualitatively similar to those shown in Fig. 3; see^34^ for all simulation details.

**Figure 4:**
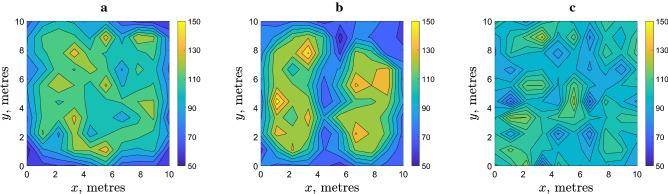
The spatial distribution of $N=10^4$ slugs at $t=10,000$ simulated for different values of the threshold density: (**a**) $d=80$, (**b**) $d=100$ and (**c**) $d=120$ (slugs/m$^{-2}$). Other parameters are as in Fig. 2.

We now recall that, while the movement parameters in Eqs. (1–5) are determined from the field data with a sufficient accuracy, the value of threshold density *d* where the movement type switches is only roughly estimated. Therefore, the next step is to investigate whether the formation of a patchy spatial distribution is sensitive to the threshold density. For this purpose, simulations were run with a different value of *d*. The results are shown in Fig. 4. We observe that the variation in *d* will not eliminate the heterogeneity, a distinctly patchy spatial distribution develops for all values of *d* used in Fig. 4. The shape and size of the patches (as is readily seen from the visual inspection of the spatial distributions) as well as the difference between the maximum and minimum values of the population density varies slightly for different *d* without showing a clear tendency. Patchiness appears to be robust to the value of density threshold in a broad range of *d* (see also Fig. 9 below), unless the average slug density is much smaller than the density threshold; in this case, slug movement is always density independent and distinct patches never form (apart from purely stochastic fluctuations of a small magnitude, cf. Fig. 3e,f).

**Figure 5:**
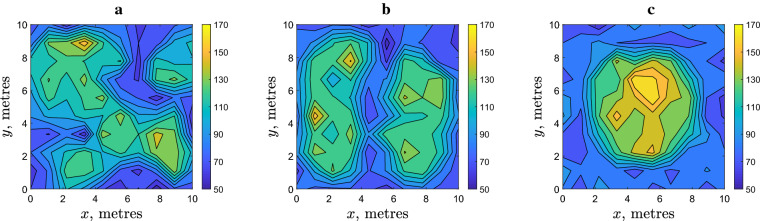
The spatial distribution of $N=10^4$ slugs at $t=10^4$ simulated for different values of the perception radius: (**a**) $R=0.5$, (**b**) $R=1$ and (**c**) $R=3$. Other parameters are as in Fig. 2.

A similar question arises about the effect of the perception radius, which value is only roughly estimated. Figure 5 shows the results obtained for different *R*. We observe that a distinct patchy structure emerges for values of *R* over a broad range, which includes the range estimated from the field data. However, contrary to the density threshold, the degree of spatial heterogeneity clearly depends on *R*. The typical size of the patches tends to increase with *R* while the number of the patches decreases accordingly. For a sufficiently large *R*, a single high density patch is formed, cf. Fig. 5c. This effect of the increase in *R* can be explained as follows. The perception radius is, by its definition, the distance within which slugs react to each other by slowing down their movement. It is a characteristic length of the population distribution, with the meaning similar to the correlation length. Slowing down of slug movement eventually leads to their numbers building up at the scale consistent with that characteristic length. Note that the average radius of the single patch shown in Fig. 5c is about 2–3 m, and this is consistent with the used value $R=3$.

### Patchiness quantification

We now complement the visual inspection of the patchy pattern with a more quantitative assessment. There are several measures or indices that are used in statistical ecology for this purpose^35^. In particular, the Morisita index^36^ $I_M$ provides a measure of how likely two individuals randomly selected from a given spatial domain are found within the same bin (e.g., quadrat) compared to that of a random distribution. It can be shown^37^ that $I_M=1$ if the individuals are distributed randomly (with a constant probability density) and $I_M>1$ if the individuals are aggregated for reasons other than purely statistical ones. The Morisita index has been widely used to quantify the heterogeneity of the spatial distribution^38–40^. It can be calculated as follows:

8 $$\begin{aligned} I_M = \frac{Q}{N(N-1)}\sum _{k=1}^{Q}n_k(n_k-1), \end{aligned}$$

where $n_k$ is the number of individuals in the *k*th bin, Q is the total number of bins (quadrats) and *N* is the total number of individuals.

**Figure 6:**
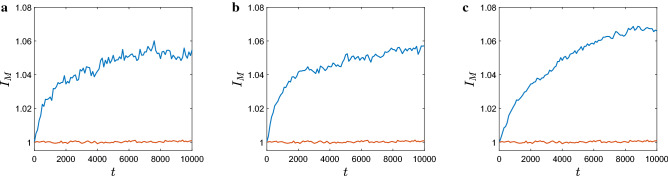
The mean Morisita Index from 10 simulation runs for different number of slugs: (**a**) $N=2.5\cdot 10^3$, (**b**) $N=5\cdot 10^3$ and (**c**) $N=10^4$. Here $R=200$ and $d=10$, other parameters are as in Fig. 2. The red curves show the Morisita index obtained in the corresponding cases of a purely random individual movement, i.e., without any density dependence.

Figure 6 shows the Morisita index calculated at each time step for a few cases with a different total number of slugs. Note that, since the movement of any individual slug is a stochastic process, $I_M$ is a stochastic quantity. In order to make sure that any tendency in $I_M$ to change is not obscured by random fluctuations (which can be of considerable amplitude), Fig. 6 shows $I_M$ averaged over ten simulation runs.

It is readily observed that, in each case shown in Fig. 6, starting from $I_M=1$ at $t=0$ (which corresponds to our choice of a uniform random initial distribution), the Morisita index then shows a clear tendency to increase on average (apart from the random fluctuations) until approximately $t=8000$ when it stabilizes at a certain value $I_M^*>1$. We therefore conclude that (i) the spatial patterns obtained in our model are self-organized, i.e. caused by interactions between the individual slugs and not by purely random, statistical effects, and (ii) in the course of time, the system reaches a dynamical equilibrium so that the Morisita index stops growing. For comparison, the red curves in Fig. 6 show the Morisita index obtained in the corresponding cases of purely random density independent movement when no high density patches are formed (cf. Fig. 3e,f).

Note that the Morisita index tends to increase slightly with an increase in the average slug density (i.e., for a fixed size of the spatial domain, with an increase in the total number of slugs *N*). Indeed, the value $I_M^*$ at which the patchiness stabilizes after a long time period rises somewhat with an increase in *N*, cf. Fig. 6a–c.

In order to provide an overall description of the emerging heterogeneous distribution, with a focus on the density dependence of the properties of the emerging patchy pattern, we use the Taylor’s Power Law aggregation index^25^. It is well known^41^ that, for populations of many different species, the mean (*m*) and the variance (*v*) of population numbers in a sample are not independent but related by a power law:

9 $$\begin{aligned} v = \alpha m^{\beta }, \end{aligned}$$

where $\alpha$ is a coefficient and exponent $\beta$ is called the aggregation index. In the case where a species has a uniform spatial distribution, $\beta$ tends towards zero; for a purely random distribution (e.g., described by Poisson distribution), $\beta =1$. Values $\beta >1$ reflect progressively greater aggregation, i.e. formation of patches in the field resulting from self-organized, density dependent dynamics of the system.

By writing Eq. (9) on the logarithmic scale:

10 $$\begin{aligned} \log (v)=\alpha + \beta \log (m), \end{aligned}$$

values of $\alpha$ and $\beta$ can be established by fitting (10) to relevant data; in particular, the aggregation index $\beta$ is defined as the slope of the regression line.

**Figure 7:**
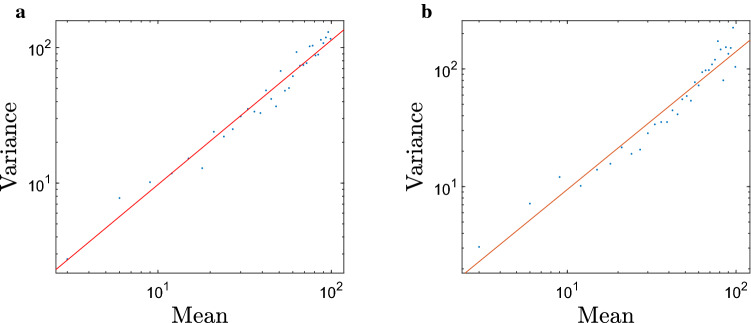
The variance of bin populations plotted against the mean bin population on a grid of 100 bins shown on a log-log scale. Each point is calculated from a single simulation and the total population is varied between simulations from $N=300$ to $N=9900$ in intervals of 300. The density dependent parameters are $d=1$ (equal to the average slug density) and $R=2$. (**a**) $t=1000$, the regression equation (10) is $\log (v)=1.066\log (m)-0.1774$, $r^2=0.9686$, (**b**) $t=10,000$, $\log (v)=1.173\log (m)-0.4551$, $r^2=0.9427$.

Figure 7 shows the aggregation index calculated for the patchy spatial patterns obtained in simulations. Recall that, when starting with a random uniform distribution, it takes a certain time for the patchy structure to develop. Correspondingly, in each simulation, the system was allowed to evolve over a certain time $t^*$ before the population was binned and the mean and the variance of the distribution were calculated. The (a) and (b) panels in Fig. 7 are obtained for $t^*=10^3$ and $t^*=10^4$, respectively. We readily see that in both cases $\beta >1$ ($\beta =1.066$ and $\beta =1.173$, respectively) confirming the self-organised, inherent nature of the spatial patterns. Note that $\beta$ is larger in Fig. 7b, that is for a larger $t^*$, which is consistent with an earlier observation that the patchy structure becomes fully developed by $t\sim 8000$.

### Evaluating trap counts

In the above, we have shown that our IBM model parameterized using field data on individual slug movement produces a distinctly heterogeneous, patchy spatial distribution. The degree of aggregation, both in terms of the Morisita index and Taylor’s aggregation index is higher than it would be due to purely stochastic reasons. The emerging patchy structure is self-organized in the sense that it emerges not due to the effect of external factors but due to an inherent property of the system such as the density dependent slug movement.

The simulated spatial patterns exhibit properties similar to the distribution of slugs in the field, in particular showing similar values of the aggregation index, which in the field data was found^18^ to be in the range $1.09<\beta <1.52$. There is, however, one difference: while the aggregation of slugs in the field is considered using trap counts, in our simulations we calculated the total size of the slug population in each bin. Obviously, the latter is considerably larger than the former, as in reality only a small fraction of the total population is caught by traps. This explains the difference (almost by an order of magnitude) between numbers shown on the colour bar in Fig. 1 (a typical example of field data) and the numbers shown on the colour bar in Figs. 2, 3, 4, 5 (simulation results).

**Figure 8:**
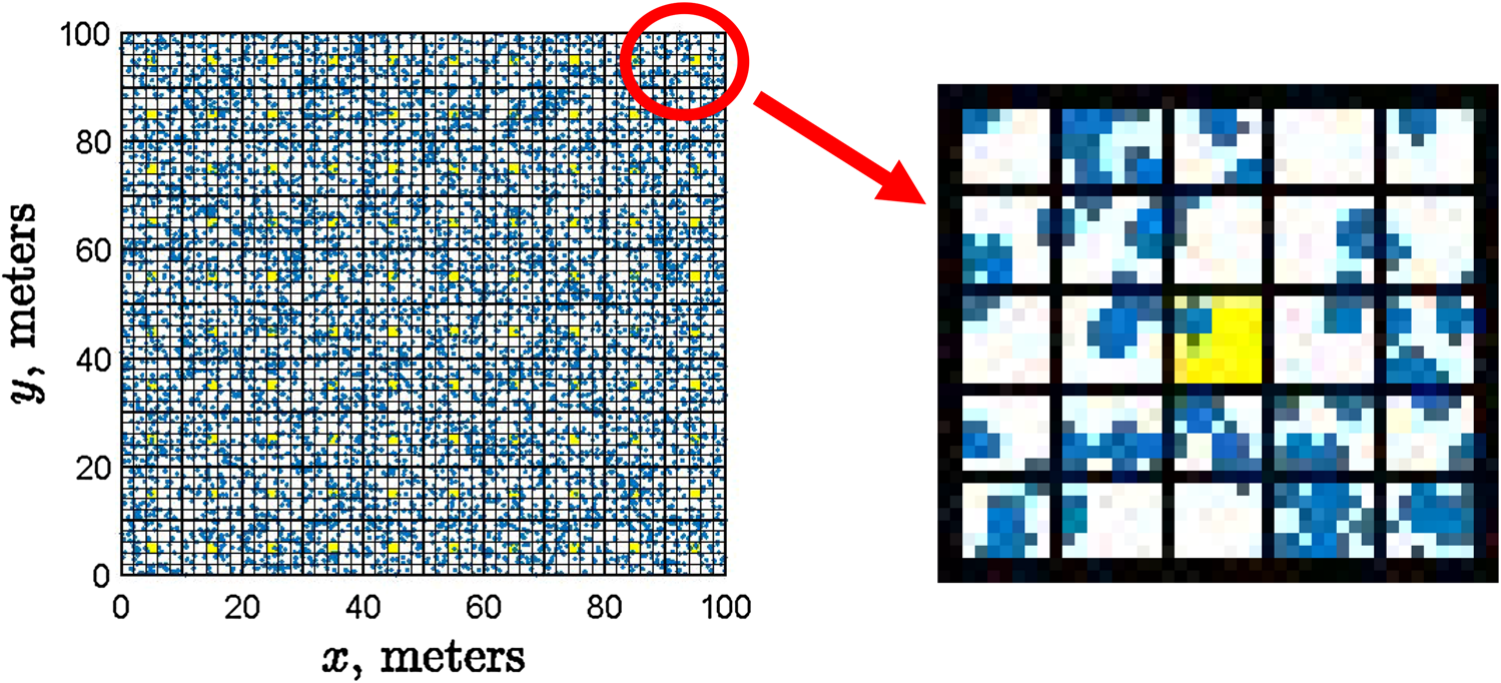
Simulating trap counts. Each bin in the $10\times 10$ grid is additionally split into 25 cells, the central cell (highlighted in yellow) being regarded as the trap’s catchment area. As an example, the right-hand side of the figure shows the magnified structure of the top-right bin of the study domain. Blue dots show the position of individual animals. The number of animals in the central cell is used as a proxy for the trap counts.

In order to resolve this discrepancy, one has to evaluate the trap counts arising from a given population density distribution in the area around the trap. This is a difficult problem, as it depends on the trap design and also requires a good understanding of fine details of animal behaviour. Reliable models to calculate trap counts only exist for the basic case of a completely random Brownian movement (which is not the case that we consider in this study) and for pitfall-type traps^42–44^, i.e. the kind of trap where, once an animal is caught, it is removed from the field. However, the design of refuge traps used in the related field study^18,24^ to which we endeavour to link our results was different. Slugs typically remain in a refuge trap for a variable period of time, but they are not removed from the field and can leave the trap at any point. Detailed modelling of trap counts arising for this trap design (e.g. taking into account a variety of slug behavioural responses) requires a separate study and hence lies beyond the scope of this paper. For the purposes of this paper, we use a different, semi-empirical approach to evaluate trap counts. Namely, we take into account that the majority of animals caught by a trap over a given exposition time come from a certain catchment area^42,43,45^, so that the closer is the position of a given animal to the trap, the larger is the probability for this animal to enter the trap. Note that, for a simpler (Brownian) movement pattern, the existence and effect of catchment area was earlier shown using rigorous mathematical arguments^43^. We assume that, for a sufficiently small catchment area, all animals end up in the trap. In order to simulate the catchment area, each of the bins in $10\times 10$ grid is split to a number of ‘sub-bins’ or cells of a smaller size (see Fig. 8). We then interpret the total number of animals in the central cell as the trap count.

**Figure 9:**
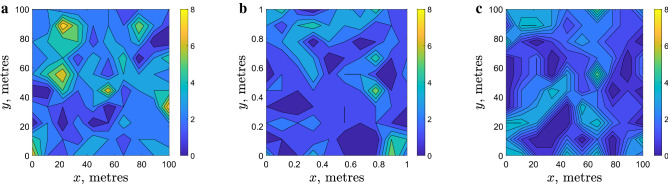
The spatial distribution of simulated trap counts (the population size in the central cell, see details in the text) obtained for $N=10^4$ slugs for different parameters of density dependence: (**a**) $d=10$, $R=1$, (**b**) $d=1$, $R=1$ and (**c**) $d=10$, $R=2$. Other parameters are as in Fig. 2.

The spatial distribution of trap counts simulated as described above in the case of 25 cells for each bin in the $10\times 10$ grid (equivalent to the grid of traps used in the field) is shown in Fig. 9. Trap counts are calculated after the initial distribution was allowed to evolve over $t=10^4$ to ensure that a distinct patchy structure emerges. It is readily seen that the simulated trap counts correspond well to those collected in the field; see Fig. 1 and also^18,19^ for more examples of field data (in particular, the supplementary material in^18^ contains data on 167 field visits).

**Figure 10:**
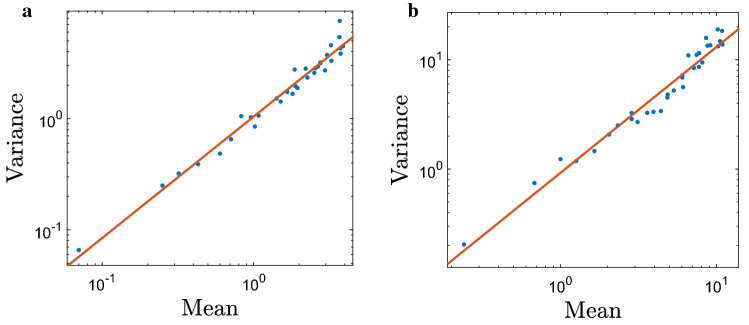
The trap count variance plotted against the mean trap count (shown on a log-log scale) calculated using the central sub-bin in each of 100 large bins for two different number of sub-bins. (**a**) $5\times 5$ cells: $\log (v) = 1.091\log (m)+0.4007$, $r^2 = 0.9739$; (**b**) $3\times 3$ cells: $\log (v) = 1.151\log (m)-0.07845$, $r2 = 0.9636$. Slug movement parameters are the same as in Fig. 7. Before calculating the binned population numbers, the initial spatial distribution was run for $t=10^4$ time steps to ensure that the emergence of a fully developed patchy structure.

A question arises here regarding whether the aggregation index is sensitive to the characteristics of the proxy used for local slug abundance, i.e. using the total number of animals in each bin or only the ‘trap count’ in its central cell. The relation between the mean and the variance of trap counts obtained in the case of $5\times 5$ cells is shown in Fig. 10a. We obtain that, in this case, the aggregation index is approximately 1.09, a slightly lower value than in the case with no splitting (cf. Fig. 7), albeit clearly larger than one.

In order to check whether the results are sensitive to the size of the catchment area (i.e. to the number of cells in each bin), we consider an intermediate case of a larger trap catchment area (i.e. splitting to a smaller number of cells). Figure 10b shows the relation between the mean and the variance of trap counts obtained in the case of $3\times 3$ cells. It is readily seen that the results are similar between the two cases and similar to the baseline case with no splitting (cf. Fig. 7). The aggregation index is consistently larger than one, being approximately between 1.09 and 1.17 for all cases considered, hence indicating a self-organized patchiness (irreducible to purely statistical reasons) in a good agreement with the field data.

## Discussion

For many species, their spatial population distributions are usually distinctly heterogeneous even in a relatively uniform environment. A number of factors and processes have been suggested as a possible explanation for this ubiquitous heterogeneity, e.g. see^3,46^ for a brief review, their applicability depending on the spatial and temporal scales of the phenomenon. On a multi-generation time scale, formation of spatial patterns is often linked to the interplay between the population dispersal and the local intra- and interspecific interactions^5–7,10^. On a shorter, within-generation time scale, one well known mechanism behind heterogeneous spatial distribution of animal species is the social behaviour resulting in the formation of mobile animal groups (swarms, flocks, etc.)^14,15^. A fingerprint of this mechanism is a high correlation between the individual velocities of the group members^16^.

In this study, based on the field data collected in our recent 3-year project focused on distribution and movement of the grey field slug^18,19,22–24^, we identified a different, new mechanism resulting in a strongly heterogeneous ‘patchy’ spatial distribution. This mechanism neither relies on a correlation between individual velocities nor results in such correlations. Patches of high animal abundance emerge as a result of a specific movement’s density dependence, i.e. slowing down of individual movement when con-specifics are present nearby, because of increasing rest times and a more uniform distribution of the turning angle increasing the path’s tortuosity. The mechanism is investigated in detail using a novel individual-based model that is parameterized using the field data. It is shown in simulations that the density dependent slug’s movement behaviour leads to formation of a patchy population distribution. The emerging spatial distributions have the properties closely resembling those observed in the field, in particular (i) exhibiting similar values of the aggregation index and (ii) showing the similar rate of temporal changes whereby patches may persist in arable fields for long periods, many throughout a cropping season.

In a broader context, our findings fall into the widely accepted paradigm stating that ecological systems are inherently multi-scale and a comprehensive understanding of a phenomenon occurring on a large scale often requires understanding of processes acting on a much smaller scale^1^. Our study provides new evidence of the linkage between different scales. The field-scale patterns in the slug abundance (with a typical size on the order of 10 m) are explained by relating them to individual slug movement that occurs on the spatial scale of about hundred times smaller (on average, tens of centimeters^22^).

A question arises about the generality of the new pattern formation mechanism. It immediately evokes another question as to how typical is the situation where the presence of con-specifics inhibits the individual movement of each animal, as it is the main prerequisite for our mechanism to act. This is an old problem, originally considered for humans in a broader context^47,48^ but more recently extended to animals. It appears that the animal response is species-specific. While for some species, e.g. see^49,50^, the presence of con-specifics tends to increase animal’s movement rate and its performance more generally (without any direct interference or collective movement), for others, e.g. some avian species^51^, it is exactly opposite. The existing studies only address a handful of species; for many species, there is no information at all. However, patches of high animal abundance are often observed in ground-dwelling invertebrate species, e.g. carabid beetles^52^ and flatworms^53,54^. Although any evidence about their social response to conspecifics is lacking, one can hypothesize that the heterogeneous distributions observed for those species arise due to the same mechanism.

This paper aims to demonstrate that the small-scale density dependent slug movement can explain the observed field-scale patterns in the slug distribution, hence relating the field data on two different spatial scales. Correspondingly, the parameter values of our individual-based model used in simulations were chosen in the range consistent with field observations. The distributions of step size and turning angle and the probability of moving are obtained from the field data unambiguously^23^; for the parameters where field data do not provide their value with satisfactory precision, such as *R* and *d*, we check the effect of their variation over a reasonable range. A question can arise here as to what are the properties of the model (e.g. the properties of the emerging patterns, if any) in the case where all parameters are varied over a much broader range. This might help to shed light on pattern formation mechanisms more generally. Investigation into this issue would reveal a detailed structure of the 6-dimensional parameter space of the model. Although beyond the scope of this paper, it should become a focus of future research.

Another question that our study leaves open is how slugs actually detect the presence of the con-specifics in their perception range. One option is an olfactory mechanism as it was found to play a role in navigation of another slug species^55^, although there is no definitive evidence that it is employed by the grey field slug. An alternative possibility which has been recorded for several slug species^56–58^ is following the slime trail, although this occurs less frequently (8% of trails) in *D. reticulatum* than in other species. Arguably, slug response to the presence of con-specifics can be somewhat different depending on the perception details: for instance, slime trail following may create a correlation in the movement direction (but not necessarily in the movement speed) while detection over short distances through volatile chemicals is more isotropic and less likely to bring any correlation. More biological evidence is needed to choose between these or other as yet unrecognised mechanisms.

## Supplementary Information

Supplementary Information.
