# Supplementary material for: A predictive model and a field study on heterogeneous slug distribution in arable fields arising from density dependent movement
Source: Sci Rep. 2022 Feb 10;12:2274. doi: 10.1038/s41598-022-05881-w (PMC8831509; doi:10.1038/s41598-022-05881-w)
Supplement: Supplementary file 1 — Supplementary Information. [file 41598_2022_5881_MOESM1_ESM.pdf]

A Predictive Model and a Field Study on Heterogeneous Slug Distribution in Arable Fields Arising from Density Dependent Movement

S. Petrovskii, J. Ellis, E. Forbes, N. Petrovskaya, K.F.A. Walters

A Supplementary Material

A.1 Morisita index

The Morisita index is known to be somewhat sensitive to the bin (quadrat) size and/or to the sample size (total number of quadrats) [1, 2]. In order to check the degree to which the choice of quadrat size (l) can affect our results, we repeated simulations for several different sizes. Note that, since the overall size of the computational domain (L) is fixed, the quadrat size and the total number of quadrats (Q) are related by the following formula:

$$Q = \left(\frac{L}{l}\right)^2, \tag{A.1}$$

(assuming that the computational domain has square shape) so that a change in l determines the change in Q.

Results are shown in Fig. A.1. We therefore observe that, although the values of the Morisita index are indeed affected by the quadrat size, the variation is relatively small, within 10% for all checked values. Thus, the results presented in Fig. 6 in the main text are typical and representative.

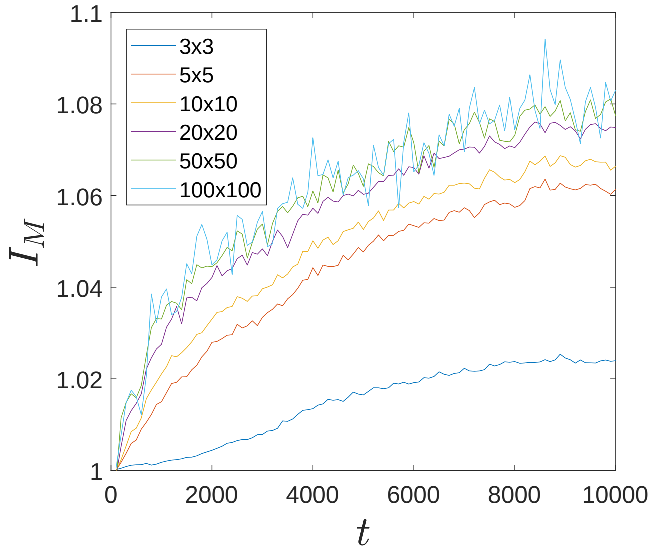

Figure A.1: The Morisita index calculated for a different quadrat size.

A.2 Long-term simulations

In order to check whether the spatial distribution converges, in the large-time limit, to a stationary distribution, we perform simulations up to $t = 10^5$, i.e. ten-fold compared to those shown in Fig. 3 in the main text. The results are shown in Fig. A.2. We readily observe that the distribution does not seem to tend to any stationary limit, as the patches are dynamic and neither their shape nor position become fixed. Given the stochastic nature of our individual-base movement model, this is perhaps not surprising. By visually comparing the distributions obtained at different times (note the variable time interval between the panels in Fig. A.2), one can conclude that, for parameter values used in simulations, the characteristic timescale of the patch dynamics is on the order of $t \sim 10^4$. Indeed, while the patterns shown in panels (e) and (f) (separated by the interval of 10^3) are quite similar, there is much less similarity between panels (b), (c) and (f) (separated by the interval of 10^4), although some patches do preserve their position. This estimate agrees well with an estimate made basing on the patch dynamics observed at earlier time, see the discussion of Fig. 3 in Section 4.1 of the main text.

Figure A.3 shows the Morisita index calculated for the same simulation run that is shown in Fig. A.2. It is readily seen that, apart from fluctuations of a purely stochastic origin, the value of the Morisita index tends to stabilize in the course of time, reaching its asymptotic value (≈ 1.065) at about $t = 3 \cdot 10^4$. This stabilization indicates that the system

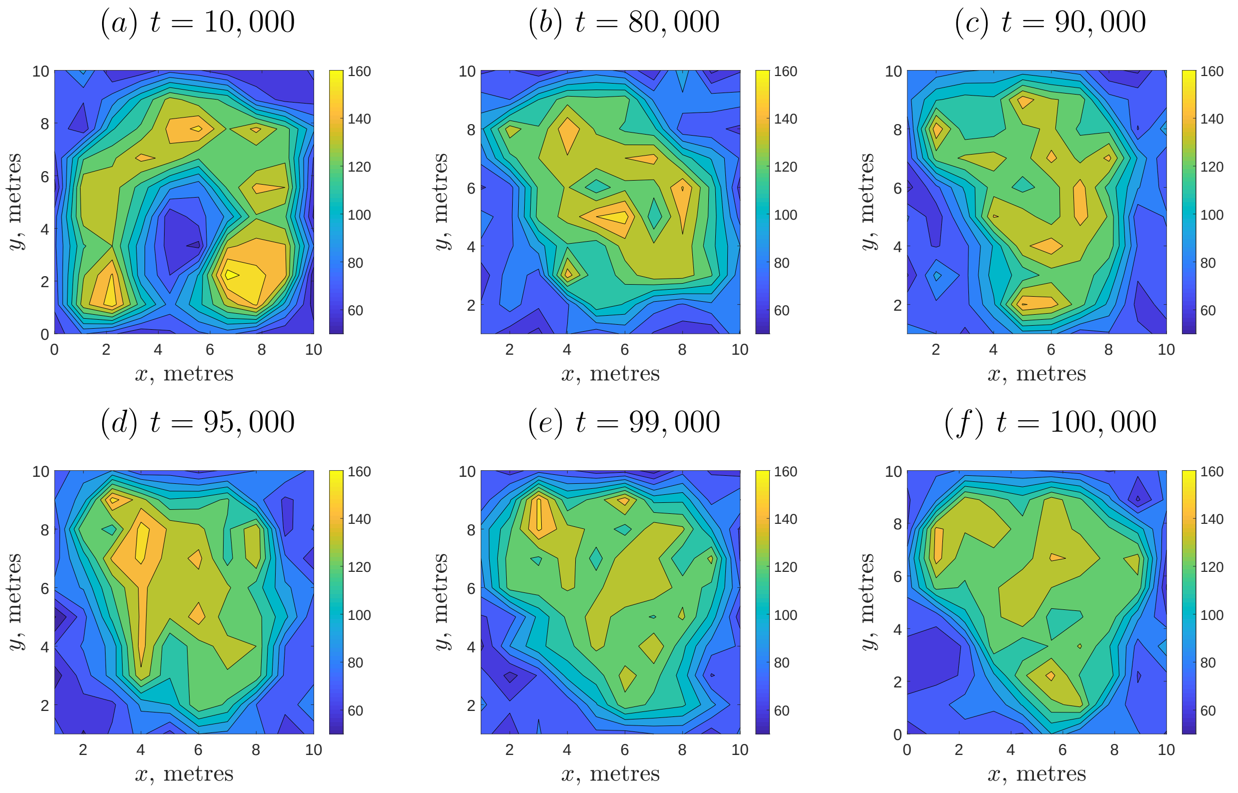

Figure A.2: The spatial distribution of $N = 10^4$ slugs shown at various moments of time (in dimensionless units, see the end of Section 3 in the main text) in a long-term simulation run. Parameter values are the same as in Fig. 3 in the main text.

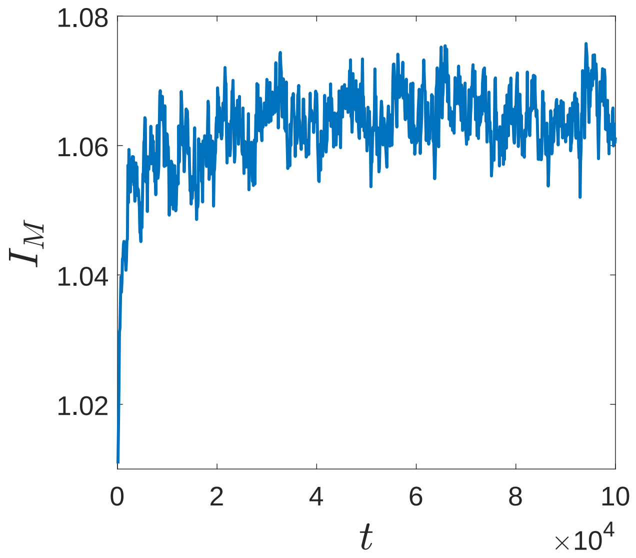

Figure A.3: The Morisita index as a function of time calculated on a longer time scale. Parameters are the same as in Fig. A.2.

approaches its equilibrium dynamics: while the patches remain dynamic, the statistics of their spatial arrangement (as is quantified by the Morisita index) stops evolving. The stochastic fluctuations around the asymptotic value indicate the dynamic nature of the patches.

Note that, contrary to Fig. 6 in the main text, the graph in Fig. A.3 is obtained for a single simulation run (there is no averaging over several runs); this explains the stochastic fluctuations of a larger amplitude compared to Fig. 6.

A.3 Computer code

The computer code used in our simulations is made freely available at:
https://github.com/DrJREllis/Density-Dependent-Slugs

Any use of the code should be explicitly acknowledged, in particular in any publication where the simulation results obtained with this code are used.

References

[1] M. Kanevski, *Analysis and Modelling of Spatial Environmental Data* (EPFL Press, Lausanne, 2004).

[2] J. J. Hayes, O. Castillo, A new approach for interpreting the Morisita index of aggregation through quadrat size. *Int. J. Geo-Inf.* **6**, 296 (2017).
